# Ultrasensitive UPLC-MS-MS Method for the Quantitation of Etheno-DNA Adducts in Human Urine

**DOI:** 10.3390/ijerph111010902

**Published:** 2014-10-21

**Authors:** Shiwei Cui, Haibin Li, Shaojia Wang, Xiao Jiang, Shusheng Zhang, Rongjie Zhang, Xin Sun

**Affiliations:** 1 Key Laboratory of Chemical Safety and Health, Chinese Center for Disease Control and Prevention, Beijing 100050, China; E-Mails: csw3068@163.com (S.C.); 18210171876@139.com (H.L.); wangsj218@sina.com (S.W.); yayafairyjiang@163.com (X.J.); 2 National Institute of Occupational Health and Poison Control, Chinese Center for Disease Control and Prevention, Beijing 100050, China; 3 Department of Chemistry, Zhengzhou University, Zhengzhou 450001, Henan province, China; E-Mail: zsszz@126.com; 4 College of Public Health, Hebei United University, Tangshan 063009, Hebei province, China; 5 Henan Center for Disease Control and Prevention, Zhengzhou 450016, Henan province, China; E-Mail: zhangrj@hncdc.com.cn

**Keywords:** UPLC-MS/MS, 1,*N*^6^-ethenodeoxyadenosine, 3,*N*^4^-ethenodeoxycytidine, lipid peroxidation, human biomonitoring

## Abstract

Etheno-DNA adducts are generated from the metabolism of exogenous carcinogens and endogenous lipid peroxidation. We and others have previously reported that 1,*N*^6^-ethenodeoxyadenosine (εdA) and 3,*N*^4^-ethenodeoxycytidine (εdC) are present in human urine and can be utilized as biomarkers of oxidative stress. In this study, we report a new ultrasensitive UPLC-ESI-MS/MS method for the analysis of εdA and εdC in human urine, capable of detecting 0.5 fmol εdA and 0.3 fmol εdC in 1.0 mL of human urine, respectively. For validation of the method, 20 human urine samples were analyzed, and the results revealed that the mean levels of εdA and εdC (SD) fmol/µmol creatinine are 5.82 ± 2.11 (range 3.0–9.5) for εdA and 791.4 ± 328.8 (range 116.7–1264.9) for εdC in occupational benzene-exposed workers and 2.10 ± 1.32 (range 0.6–4.7) for εdA and 161.8 ± 200.9 (range 1.8–557.5) for εdC in non-benzene-exposed workers, respectively. The ultrasensitive detection method is thus suitable for applications in human biomonitoring and molecular epidemiology studies.

## 1. Introduction

Etheno-DNA adducts 1,*N*^6^-ethenodeoxyadenosine (εdA) and 3,*N*^4^-ethenodeoxycytidine (εdC) are formed not only from exogenous carcinogens vinyl chloride and urethane [1,2,3,4], but also peroxidation of arachidonic acid and liver microsomal membranes in the presence of LPO-inducing compounds [5,6,7]. These miscoding lesions are elevated in various diseases, such as Wilson’s disease and primary hemochromatosis induced by excess metal storage or chronic inflammation and infections [8,9]. A very high ω-6 polyunsaturated fatty acid diet strongly increased etheno-DNA adduct levels in white blood cells, particularly in female subjects [10]. Mice injected with RcsX cells, which cause overexpression of inducible nitric oxide synthase (iNOS), exhibited six-fold higher etheno adduct levels in the spleen DNA compared with the controls [11]. Etheno-DNA adducts were also found in the affected tissue of chronic pancreatitis patients [12]. Our recent study found that 4-hydroxy-estradiol metabolite formation and high ω-6 PUFA intake were both linked to increased formation of LPO-derived adducts in white blood cells of premenopausal women [13]. A recent study first detected the presence of such etheno-modified 5mdC residues (ε5mdC) in human tissue DNA [14]. These observations suggested that etheno-DNA adducts accumulate in cancer-prone tissues as a result of chronic inflammatory processes causing oxidative stress and LPO.

Non-invasive detection methods, such as urinalysis, will expedite studies in humans aimed to elucidate etiopathological factors that cause oxidative DNA damage. For these reasons, Nair and others have established methods to study the formation and excretion of etheno-DNA base adducts; εdA and εdC were found to be present in human urine, and these could be biomarkers for DNA damage produced by persistent oxidative stress and lipid peroxidation [15,16,17]. The method, involving immunoprecipitation, high-performance liquid chromatography and fluorescence detection, has been applied to evaluate the effects of some dietary factors on the formation of urinary εdA in non-smoking post-menopausal women [18]. Chen *et al.* developed a gas chromatography/negative ion chemical ionization/mass spectrometry (GC/NICI/MS) method for the detection of 3,*N*^4^-ethenocytosine (εC) and 1,*N*^6^-ethenoadenine (εA) in human urine [19,20]. A liquid chromatography-electrospray ionization-tandem mass spectrometry (LC-ESI-MS-MS) method had been developed for urinalysis compared with a GC/NICI/MS method [21]. We developed a ^32^P-postlabeling/TLC method for analysis of εdC using a multi-substrate deoxyribonucleoside kinase enzyme in small amounts of human urine [22]. The present method, although highly sensitive, requires the use of a radioisotope or considerable time and effort.

We now report a new ultra performance liquid chromatography-electrospray ionization-tandem mass spectrometry (UPLC-ESI-MS/MS) method for the analysis of εdA and εdC adducts in human urine. For validation of the method, 20 human urine samples randomly selected from female workers who participated in a cross-sectional epidemiology study of occupational benzene exposure were assayed, and the results indicate that our ultrasensitive method appears to be superior to other methods reported in terms of: (1) high sensitivity and specificity; (2) low amounts of urine sample required; (3) capability to detect background levels of etheno-DNA adducts in human biological samples; and (4) reliable monitoring of the disease-related increase of these substances in patients. We present the details of the method protocol and its application to quantify εdA and εdC in human urine samples.

## 2. Material and Methods

[^15^N_3_]-2’-deoxycytidine and [^15^N_5_]-2’-deoxyadenosine were purchased from Cambridge Isotope Laboratories (Andover, MA, USA). 1,*N*^6^-etheno-2’-deoxyadenosine, 2’-deoxycytidine and 2’-deoxyadenosine were obtained from Sigma-Aldrich Corporation (St. Louis, MO, USA). All solvents were of HPLC grade.

### 2.1. Synthesis of Isotope Labeled [^15^N_5_]-1,N^6^-Etheno-2’-deoxyadenosine ([^15^N_5_]-εdA) and [^15^N_3_]-3,N^4^-Etheno-2’-deoxycytidine ([^15^N_3_]-εdC)

[^15^N_5_]-εdA and [^15^N_3_]-εdC were synthesized following the reported procedures [21,23,24]. [^15^N_5_]-εdA and [^15^N_3_]-εdC were eluted by a reverse phase HPLC (Agilent 1200) equipped with a Phenomenex Synergi Hydro-RP 80A column (250 × 4.6 mm, 4 μm) using an isocratic program with 5% of acetonitrile in 8 mM ammonium acetate; the flow rate was 0.5 mL/min. [^15^N_5_]-εdA and [^15^N_3_]-εdC were then purified using an isocratic program with 20% acetonitrile in water; the flow rate was 0.5 mL/min. Quantifications of [^15^N_5_]-εdA and [^15^N_3_]-εdC were performed by UV spectrometry using absorption coefficients of 10,300 M^−1^ cm^−1^ at 260 nm and 12,000 M^−1^ cm^−1^ at 272 nm, respectively, as internal standards (I.S.) [24,25,26].

### 2.2. Synthesis of Unlabeled 3,N^4^-Etheno-2’-Deoxycytidine (εdC)

Synthesis and purification of unlabeled εdC were followed by the reported procedure described above. Quantification of εdC was performed by UV spectrometry as a standard [26].

### 2.3. Preparation of Urine Samples

Preparation of urine samples followed our reported procedure with minor modifications [22]. Briefly, 1.0-mL urine samples were filtered through a 0.22-micron filter and were spiked with 100 pg of [^15^N_5_]-εdA and [^15^N_3_]-εdC as internal standards (I.S.). The urinary protein was precipitated by adding 1.5 mL cold ethanol and centrifuged at 4000 rpm for 10 min after standing at –20 °C for 30 min. The supernatant (~2.5 mL) was moved to a new tube and concentrated to dryness by vacuum centrifugation after protein precipitation. The dried urine sample was redissolved in 0.5 mL of ddH_2_O and loaded onto a C18 OH solid-phase silica column (BondElut^R^, Agilent Technologies, 500 mg, 3 mL, USA). The columns were washed with 12 mL of water followed by 3 mL of 10 % methanol (v/v) and 3 mL of 15% methanol (v/v) in order to remove the bulk of normal nucleosides. The columns were then eluted twice with 2.5 mL of 30% methanol (v/v). Before sample loading, the columns were pre-washed with 15 mL of methanol followed by 15 mL of water. The elute was concentrated to dryness by vacuum centrifugation.

### 2.4. Urinary Etheno-DNA Adduct Analysis

The dried urine sample was dissolved in 25 μL water, and 5 μL was subjected to UPLC-MS/MS for etheno-DNA adducts analysis. UPLC-MS/MS analysis was performed by UPLC (Waters ACQUITY UPLC) equipped with an ACQUITY TQD mass spectrometry connecting with a Waters ACQUITY UPLC BEH C18 column (HP 19091S-433, 2.1 × 50 mm, 1.7 μm). The sample was eluted using a linear program with a water (A)/methanol (B) gradient: 0–9 min, 5–7 % B in A; 9–15 min, 7–100% B in A; 15–18 min, 100–5% B in A; 18–20 min, 5% A, isocratically. The flow rate was 0.2 mL/min. ACQUITY TQD mass spectrometry results are given in Table 1. Positive ions were acquired in the multiple reaction monitoring (MRM) mode for transitions of the protonated εdA molecule [(M + H)^+^] (m/z 276) to m/z 160 and for the corresponding [(M + H)^+^] (m/z 281) to m/z 165, for transitions of the protonated εdC molecule [(M + H)^+^] (m/z 252) to m/z 136 and for the corresponding [(M + H)^+^] (m/z 255) to m/z 139.

For quantifications of εdA and εdC adducts, 100 pg of εdA and εdC as external standards were mixed with 100 pg of [^15^N_5_]-εdA and [^15^N_3_]-εdC as internal standards in 25 μL of the mixture; 5 μL were injected into UPLC-MS-MS in parallel. The amounts of εdA and εdC adducts were calculated as (F2/F1), whereby F1 = [the peaks of εdA and εdC adducts in standard]/[the peaks of [^15^N_5_]-εdA and [^15^N_3_]-εdC (I.S.) in standard] and F2 = [the peaks of εdA and εdC adducts in sample]/[the peaks of [^15^N_5_]-εdA and [^15^N_3_]-εdC in sample]; 100 pg of [^15^N_5_]-εdA and [^15^N_3_]-εdC were added to each urine sample as an internal standard for correcting the recovery.

For the determination of creatinine in urine samples, the automatic Cobas Integra Creatinine Enzymatic (CREAE) test was used. The test is based on an enzymatic, colorimetric method (PAP) [27].

**Table 1:** ACQUITY TQD mass spectrometry for the detection of etheno-DNA adducts in human urine by UPLC-ESI-MS/MS.

Parameters	εdA	εdC
^14^N Q1-Q3 m/z	276 → 160	252 → 136
^15^N Q1-Q3 m/z	281 → 165	255 → 139
Source Temperature (°C)	110	110
Desolvation Temperature (°C)	350	350
Desolvation (L/h)	650	650
Cone (L/h)	50	50
Capillary Voltage (kV)	3	3
Collision Voltage (V)	20	20
Collision energy (eV)	12	12
Collision Gas (mL/min)	0.12	0.12

## 3. Results

### 3.1. Urinary Analysis of Etheno-DNA Adducts

**Figure 1:**
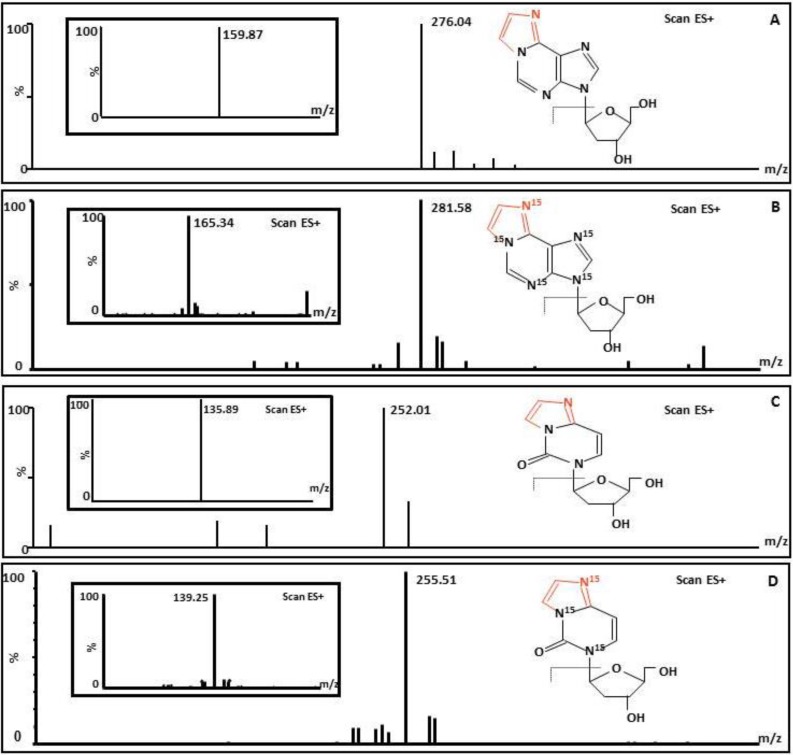
Structures and mass spectra of the 1,*N*^6^-etheno-2’-deoxyadenosine and 3,*N*^4^-etheno-2’-deoxycytidine standards and [^15^N_5_]-1,*N*^6^-etheno-2’-deoxyadenosine and [^15^N_3_]-3,*N*^4^-etheno-2’-deoxycytidine as internal standards (I.S.). (**A**) εdA; (**B**) [^15^*N*_5_]εdA; (**C**) εdC; (**D**) [^15^*N*_3_]εdC.

Purification and enrichment of εdA and εdC in urine samples were achieved using a solid-phase silica column. The fractions of εdA containing [^15^N_5_]-εdA and εdC containing [^15^N_3_]-εdC were eluted with 30% methanol in water (v/v) and then concentrated by vacuum centrifugation. Urine samples were quantified by comparing the areas of the εdA and εdC chromatogram peaks to those of the [^15^N_5_]-εdA and [^15^N_3_]-εdC chromatogram peaks. Levels of etheno-DNA adducts detected in human urine samples were given in Table 2. One hundred picrograms of [^15^N_5_]-εdA and [^15^N_3_]-εdC spiked into each urine sample as I.S. were found to be reproducible with a recovery of [^15^N_5_]-εdA of 78.3% ± 10.1 and a recovery of [^15^N_3_]-εdC of 79.3% ± 8.7. Figure 1 shows the typical structures and MS/MS characterizations of εdA and [^15^N_5_]-εdA and εdC and [^15^N_3_]-εdC using the ES ionization interface with a sources block of 110 °C and a desolvation temperature of 350 °C.

### 3.2. Reproducibility of UPLC-MS-MS Method

The UPLC-MS/MS method was validated with respect to the intra-assay and inter-assay. A control urine sample was prepared by diluting 50:50 (v/v) with a urine sample of non-benzene-exposed workers and water. The control urine sample was spiked with 100 pg of [^15^N_5_]-εdA, [^15^N_3_]-εdC as I.S. These single urine samples were prepared following adduct enrichment by loading a C18 OH solid phase silica column after protein precipitation. These single urine samples were injected into the UPLC-MS/MS at the same time for the intra-assay. The recovery level of [^15^N_5_]-εdA ranged from 71.0% to 83.0%, and the average recovery level was 75.7% ± 6.43; the recovery level of [^15^N_3_]-εdC ranged from 71.0% to 81.0%, and the average recovery level was 74.7% ± 5.51. For the inter-assay, the control urine sample was spiked with 100 pg of [^15^N_5_]-εdA, [^15^N_3_]-εdC as I.S.; these single samples were prepared following the same procedures and then injected into the UPLC-MS/MS on different days. The recovery of [^15^N_5_]-εdA ranged from 81.0% to 84.0%, and the average recovery level was 82.7% ± 1.53; the recovery level of [^15^N_3_]-εdC ranged from 79.0% to 82.0%, and the average recovery level was 80.7% ± 1.53.

**Table 2:** Levels of etheno-DNA adducts detected in human urine samples.

Code No.	Levels of Etheno-DNA Adducts (pmol/L)		Levels of Etheno-DNA Adducts (fmol/μmol Creatinine)	
	εdA	εdC	εdA	εdC
BF-1	13.4	1077.7	9.5	758.9
BF-2	5.8	1738.8	3.0	918.3
BF-3	7.8	1,312.1	7.4	1242.5
BF-4	3.8	680.0	3.2	565.9
BF-5	5.8	1163.1	5.5	1,101.4
BF-6	11.1	1133.5	8.0	819.2
BF-7	5.4	1658.1	4.1	1264.9
BF-8	11.5	1893.2	5.2	852.3
BF-9	6.8	269.6	6.9	274.2
BF-10	10.7	233.8	5.4	116.7
CF-1	7.0	15.6	2.2	4.9
CF-2	3.1	314.4	4.7	479.6
CF-3	3.2	17.5	0.6	3.1
CF-4	4.3	242.9	2.0	115.0
CF-5	2.9	155.2	4.0	213.1
CF-6	14.9	1092.9	1.3	96.2
CF-7	7.2	378.5	2.4	125.2
CF-8	4.3	9.8	0.8	1.8
CF-9	8.2	111.0	1.6	21.5
CF-10	0.8	304.5	1.5	557.5
CF-1	7.0	15.6	2.2	4.9

### 3.3. Recovery of UPLC-MS-MS Analysis

The recovery of the UPLC-MS-MS method was achieved by adding 50 pg and 100 pg of [^15^N_5_]-εdA and [^15^N_3_]-εdC in control urine samples as I.S., respectively. These six single urine samples were prepared following the same procedure and then injected into the UPLC-MS-MS for the calculation of the recovery. The recovery of [^15^N_5_]-εdA by adding 50 pg ranged from 68.0%–83.0%, and the average recovery was 78.0% ± 8.7; the recovery of [^15^N_5_]-εdA by adding 100 pg ranged from 72.0%–90.0%, and the average recovery of [^15^N_5_]-εdA was 78.3% ± 10.1. The recovery of [^15^N_3_]-εdC by adding 50 pg ranged from 84.0%–87.0%, and the average recovery of [^15^N_3_]-εdC was 86.0% ± 1.73; the recovery of [^15^N_3_]-εdC by adding 100 pg ranged from 72.0 %–89.0%, and the average recovery of [^15^N_3_]-εdC was 79.3% ± 8.74.

### 3.4. Response Curve of UPLC-MS/MS Analysis

**Figure 2:**
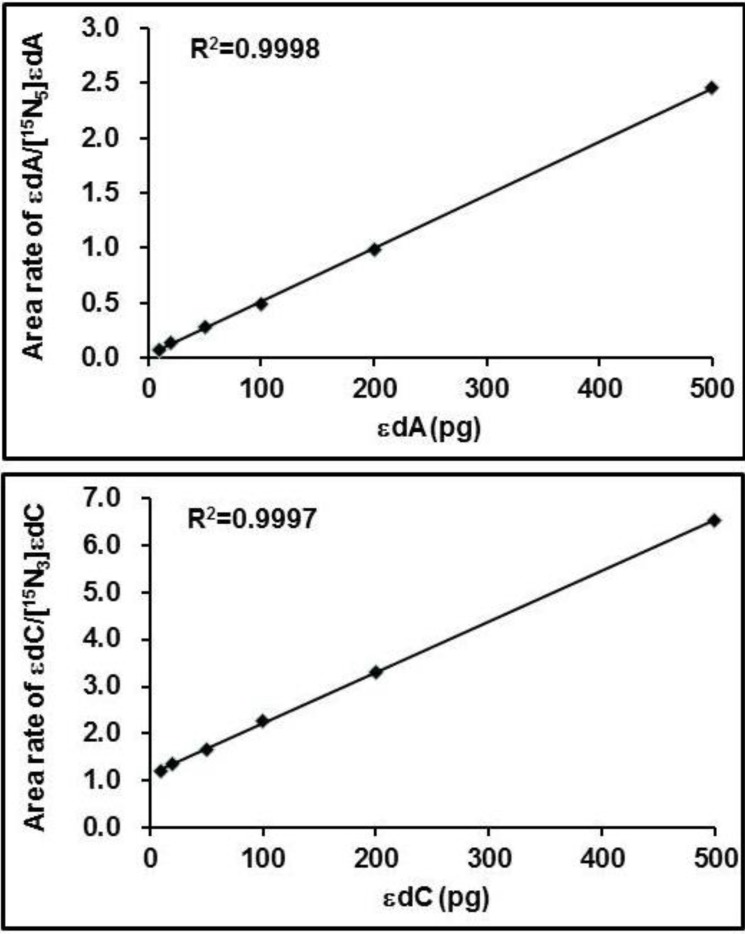
The substrate concentration-dependent response curve of the substrate (quantities of εdA and εdC) and the area ratios of εdA/[^15^N_3_]-εdA and εdC/[^15^N_3_]-εdC by UPLC-MS-MS analysis.

The substrate concentration-dependent response curve of the UPLC-MS/MS analysis was obtained by analysis of 10, 20, 50, 100, 200 and 500 pg of εdA, εdC standards spiking of 100 pg of [^15^N_5_]-εdA, [^15^N_3_]-εdC as I.S. in the control urine samples; these single urine samples were prepared following the same procedures and then were subjected to UPLC-MS/MS. There are linear correlations between the substrate (quantities of εdA and εdC) and response (peak area ratios of εdA/[^15^N_5_]-εdA and εdC/[^15^N_3_]-εdC) (Figure 2). It is estimated that the limits of detection (s/n = 3) of εdA and εdC for a 1.0-mL human urine sample are around 0.5 pM and 0.3 pM, respectively.

### 3.5. Etheno-DNA Adducts Detected in Human Urine

**Figure 3:**
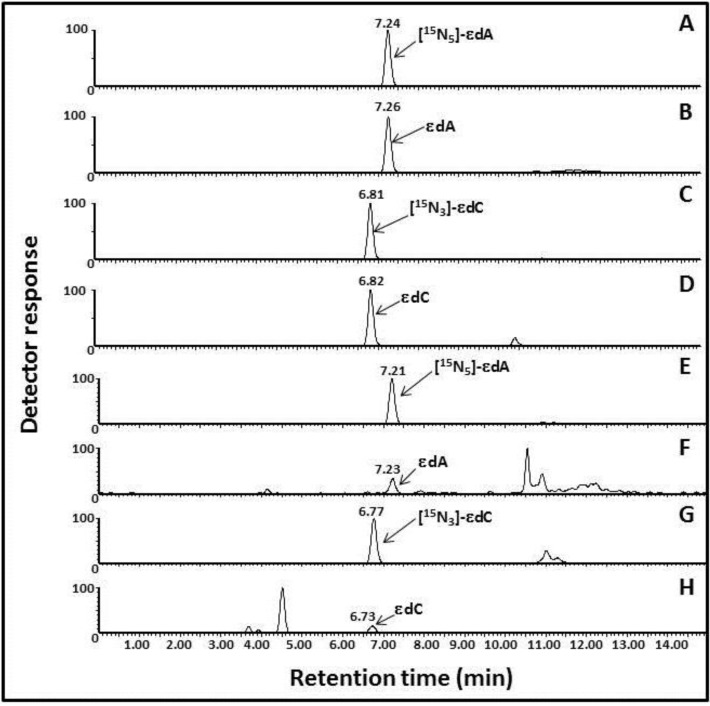
Typical UPLC-MS-MS chromatography profiles of 1,*N*^6^-etheno-2’-deoxyadenosine (εdA) and 3,*N*^4^-etheno-2’-deoxycytidine (εdC) detected in human urine. A, [^15^*N*_5_]εdA as I.S.; B, εdA standard; C, [^15^N_3_]εdC as I.S.; D, εdC standard; E, [^15^*N*_5_]εdA as I.S. in 1.0 mL of human urine; F, εdA detected in 1.0 mL of human urine; G, [^15^N_3_]εdC as I.S. in 1.0 mL of human urine; H, εdC detected in 1.0 mL of human urine.

To validate this UPLC-MS-MS method, 20 urine samples randomly selected from female workers who participated in a cross-sectional epidemiology study of occupational benzene exposure were assayed. The typical autoradiograms of εdA and εdC detected in human urine samples are shown in Figure 3. The results revealed a significant difference between levels of εdA (SD) fmol/µmol creatinine, which are 5.82 ± 2.11 (range 3.0–9.5) in female benzene-exposed (BF) workers and 2.10 ± 1.32 (range 0.6–4.7) in female non-benzene-exposed (CF) workers (*p* = 0.0015). The levels of εdC (SD) fmol/µmol creatinine are 791.4 ± 382.8 (range 116.7–1264.9) in benzene-exposed workers and 161.8 ± 200.9 (range 1.8–557.5) in non-benzene-exposed workers, for which significant differences were also found (*p* = 0.0013) (Table 3).

**Table 3:** Levels of etheno-DNA adducts (εdA and εdC) in human urine of occupational benzene-exposed and non-exposed workers (fmol/μmol creatine) *.

Etheno-DNA Adducts	Non-Exposed Workers					Occupational-Exposed Workers					*p-Value*
	No. sample ^a^	Mean ^b^	SD ^c^	Min ^d^	Max ^e^	No. sample ^a^	Mean ^b^	SD ^c^	Min ^d^	Max ^e^	
εdA	10/10	2.10	1.32	0.6	4.7	10/10	5.82	2.11	3.0	9.5	0.0015
εdC	10/10	161.8	200.9	1.8	557.5	10/10	791.4	382.8	116.7	1264.9	0.0013

^a^ Number of subjects with detectable levels/total number of subjects; ^b^ mean levels of etheno-DNA adducts (fmol/μmol creatine); ^c^ standard deviation; ^d^ minimum levels of etheno-DNA adducts (fmol/μmol creatine); ^e^ maximum levels of etheno-DNA adducts (fmol/μmol creatine); * a *t*-test was used for statistical analysis, and a *p*-value of <0.05 was considered to indicate a significant difference.

## 4. Discussion

Increased oxidative stress and lipid peroxidation are implicated in multistage carcinogenesis. Etheno adducts are formed in DNA bases after reaction with aldehydes, such as trans-4-hydroxy-2-nonenal (HNE), generated during oxidative stress as lipid peroxidation-end products. These DNA adducts are believed to be important in the etiology of cancer [1,2,3,4,5,6]. Existing methods for quantifying DNA adducts use ^32^P-postlabeling; although highly sensitive, postlabeling requires the use of an energetic radioisotope and considerable time and effort [15,22,23]. The LC-MS/MS methodology reported everywhere permits automated quantification of trace levels of DNA adducts. Therefore, we now report a new ultrasensitive UPLC-MS/MS method for the analysis of εdA and εdC adducts in human urine. Our method is based on the effective purification and enrichment of εdA and εdC in human urine samples; hereby, solid-phase silica column chromatography has been developed that allows an efficient separation of εdA and εdC adducts from the urinary matrix. The absolute sensitivities of our method were found to be 0.5 fmoles of εdA and 0.3 fmoles of εdC detectable in 1.0 mL of human urine, thus being a magnitude higher than the currently reported GC/NICI/MS method with a sensitivity of 12 fmoles of εdC in 0.1 mL urine [20]. For purification and enrichment of etheno-DNA adducts from normal nucleosides in human urine, Bond Elut LC_18_-OH (Agilent, 500 mg, 3 mL) and Oasis HLB (Waters, 220 mg, 6 mL) silica-phase columns were used, respectively, to achieve an efficient enrichment of etheno-DNA adducts from human urine. The Bond Elut LC_18_-OH silica-phase column was shown to be valid for achieving a better recovery of etheno-DNA adducts from urine matrix. For validating the reliability of our method, [^15^N_5_]-εdA and [^15^N_3_]-εdC were added to each urine sample as internal standards, allowing correction of the recovery. Results on the intra- and inter-assay variability revealed a high reproducibility and accuracy. Therefore, our ultrasensitive method appears to be superior to other methods reported in terms of: (1) high sensitivity and specificity; (2) low amounts of urine sample required; (3) capability to detect background levels of etheno adducts in human urine; and (4) high-throughput and reliable monitoring of the disease-related increase of these substances in patients.

A series of studies in animals and humans have demonstrated that etheno-DNA adducts are among the ideal markers for DNA damage produced endogenously as a result of persistent oxidative stress and lipid peroxidation (LPO) [9,10,11,12,13,14]. There is increasing evidence for a role of reactive oxygen species and lipid peroxidation in the etiology of human cancers and the development of other chronic degenerative diseases. The development and application of sensitive and specific detection methods for this class of DNA-adducts should provide valuable tools for investigating their role in human disease pathogenesis and its preventability.
